# High-Efficient FLPo Deleter Mice in C57BL/6J Background

**DOI:** 10.1371/journal.pone.0008054

**Published:** 2009-11-26

**Authors:** Yingjie Wu, Chunxin Wang, Hui Sun, Derek LeRoith, Shoshana Yakar

**Affiliations:** 1 Endocrinology/Diabetes and Bone Disease Division, Mount Sinai School of Medicine, New York, New York, United States of America; 2 Biochemistry Section, SNB/NINDS, National Institutes of Health, Bethesda, Maryland, United States of America; University of Texas MD Anderson Cancer Center, United States of America

## Abstract

Conditional gene manipulation in mice becomes a routine for genetic studies of mammalian gene functions. Additional site-specific recombinases such as FLP or φ31 provide one more level of gene manipulation flexibility. The recombination activity of the currently available FLP deleter mice remains low. We generated a new FLP deleter mouse line with the mouse codon-optimized FLPo gene in C57BJ/6 background, which showed superior recombination efficacy in comparison to FLPe deleter mice. 100% complete removal of FRT-flanked Neo cassette was observed in all F1 progeny mice carrying both FLPo and Neo cassette, which can be transmitted to F2 generation independent of FLPo activity. Our new FLPo transgenic mice (on pure C57BJ/6 background) will largely facilitate the gene targeting process and is valuable for conditional gene manipulation.

## Introduction

Gene targeting via homologous recombination remains a powerful tool for functional analyses of mammalian genes. The advent of multiple site-specific recombinases (such as Cre, FLP and φ31) allows delicate gene manipulation in a tissue- or time-specific manner. These conditional knockout (CKO) mice are not only desired when embryonic lethality caused by deletion of essential genes, but also are better models for human diseases. The Cre-LoxP system alone could be used for conditional knockout. However, removal of the selection marker from the genome by partial Cre activity in vivo is complicated, time-consuming and may be gene-dependent [1], [2]. Therefore, a combination of Cre/loxP and FLP/FRT systems became more widely used. Moreover, generation of new CKO constructs by so called “knockout-first” strategy that combines gene trap and CKO cassettes allows for the generation of multipurpose alleles (a conventional KO, a conditional KO or a reporter allele) in one targeting construct [3], which demands high efficient FLP recombinase activity in vivo to achieve such flexibility. However, as originally isolated from yeast, FLP recombinase activity is significantly reduced at 37°C due to enhanced thermal-instability. Hence, only a few studies were able to apply the FLP/FRT system in mice, but with moderate efficiency [4], [5].

Initial efforts were made to enhance the thermo-stability of FLP enzyme by random mutagenesis through molecular evolution. As a result, a new version of FLP, FLPe exhibited 4–10 fold higher activity than FLP at 37°C [6]. However, despite this improvement, the recombination efficiency of FLPe in cells and in vivo remains low with mosaic recombination found in almost all ES cell [4], [7]. Approximately 50% of double transgenic mice generated by crossing the FLPeR mice with mice carrying the FRT-disrupted lacZ-transgene had complete recombination in all adult tissues. Some double transgenic mice had mosaicism, though many of them transmitted recombined transgenes to their progenies [8]. Takeuchi et al. generated FLP deleter mouse lines that added a nuclear localization signal (NLS) at the N-terminus of the original FLP driven by a strong *EFα* promoter, and achieved up to 71% recombination efficiency in the C57BL/6N genetic background [9]. High recombination rates were observed in Hiroaki Kanki generated CAG-NLS-FLPe transgenic mice in C57BL/6J genetic background in their two lines [10]. FLPe showed limited recombination rate in ES cells with mosaic recombination in almost every clone [7]. In direct comparison, it was shown that FLPe exhibits only 25% of the efficiency of Cre on the reporter LacZ cassette and 10% efficiency of Cre on chromatin targets [11]. In our own experience, we have had very limited success with FLPe both in vivo and in vitro.

Recently, Raymond et al synthesized de novo a mouse codon-optimized FLP version, FLPo, which was shown to exhibit significantly enhanced recombinase activity comparable to Cre in ROSA26-based ES reporter cells [12]. This prompted us to generate FLPo transgenic mice, which turn out to be very efficient in removing the FRT-flanked selection marker. The new FLPo deleter mice largely facilitated our process of generating the GHR (growth hormone receptor) conditional knockout mice. The superior recombination efficacy of this FLPo deleter mouse line will be beneficial to the scientific community and particularly valuable for conditional gene manipulation in the pure C57BL/6 genetic background.

## Results

**Figure 1:**
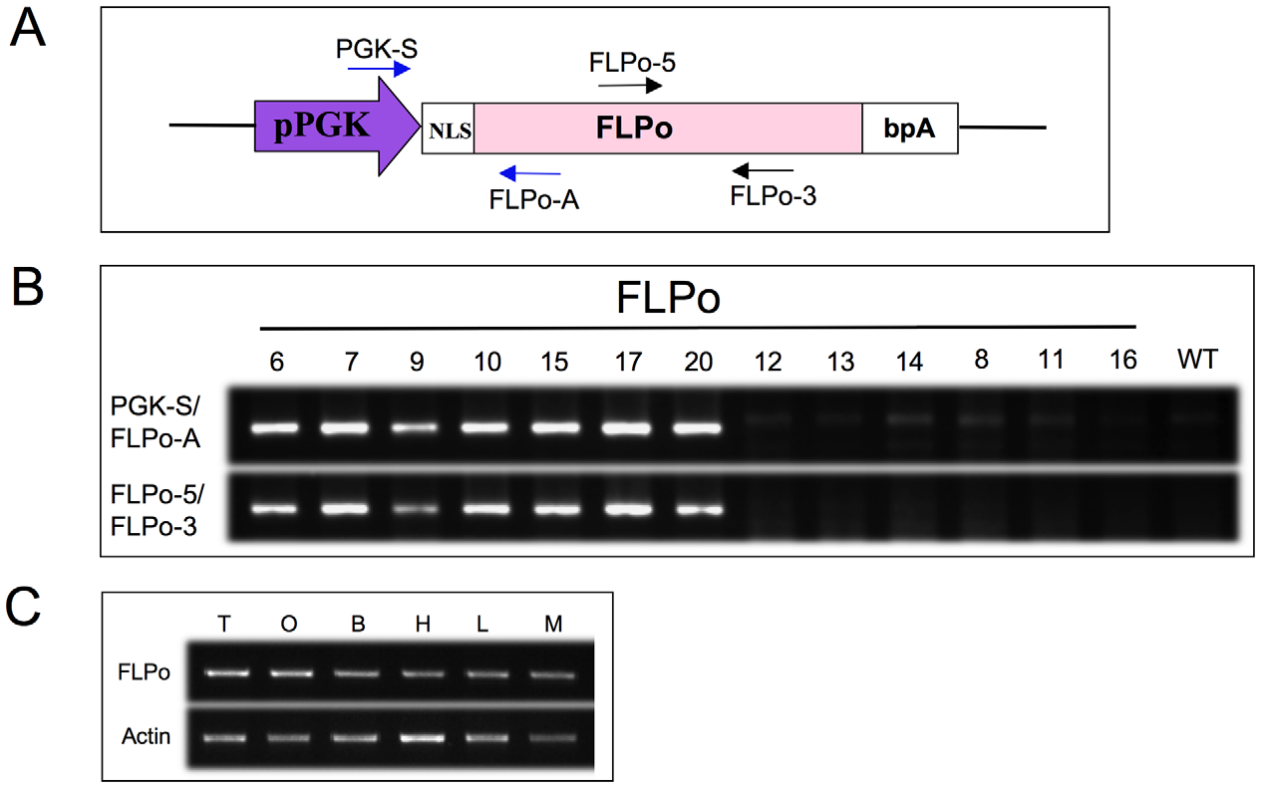
Generation of FLPo transgenic mice. (A) Schematic diagram of FLPo construct used for the generation of FLPo transgenic mice. Two primer sets for genotyping of FLPo transgenic mice are indicated. (B) PCR analysis of FLPo positive founder lines. (C) RT-PCR of FLPo mRNA in germ line [Testis (T) and ovary(O)] and other tissues [brain(B), heart(H), liver(L) and muscle(M)] of transgenic mouse FLPo-10 line. Actin was used as control.

pPGKFLPobpA expresses de novo synthesized mouse codon-optimized FLP (FLPo) under the PGK promoter (Fig. 1A), and is highly efficient in inducing recombination in mouse embryonic stem cells [12] and human HCT116 cells (unpublished data). To generate a FLPo deleter mouse line, we injected the linearized PGK-FLPobpA fragment into pronuclei of fertilized eggs of the C57BL/6 mice. Seven transgenic founder mice were identified by PCR genotyping (Fig. 1B), six of which transmitted the FLPo transgene through the germ cells. The PGK-1 promoter activates transgene expression as early as in 3.5d and in adults it drives target genes expression ubiquitously in all tissues including the gonads [13]. FLPo expression levels were analysed in various tissues, including gonads by RT-PCR. As shown in Fig. 1C, the expression of the FLPo transgene can be detected in all tissues examined with the strongest level in testis and ovary.

**Figure 2:**
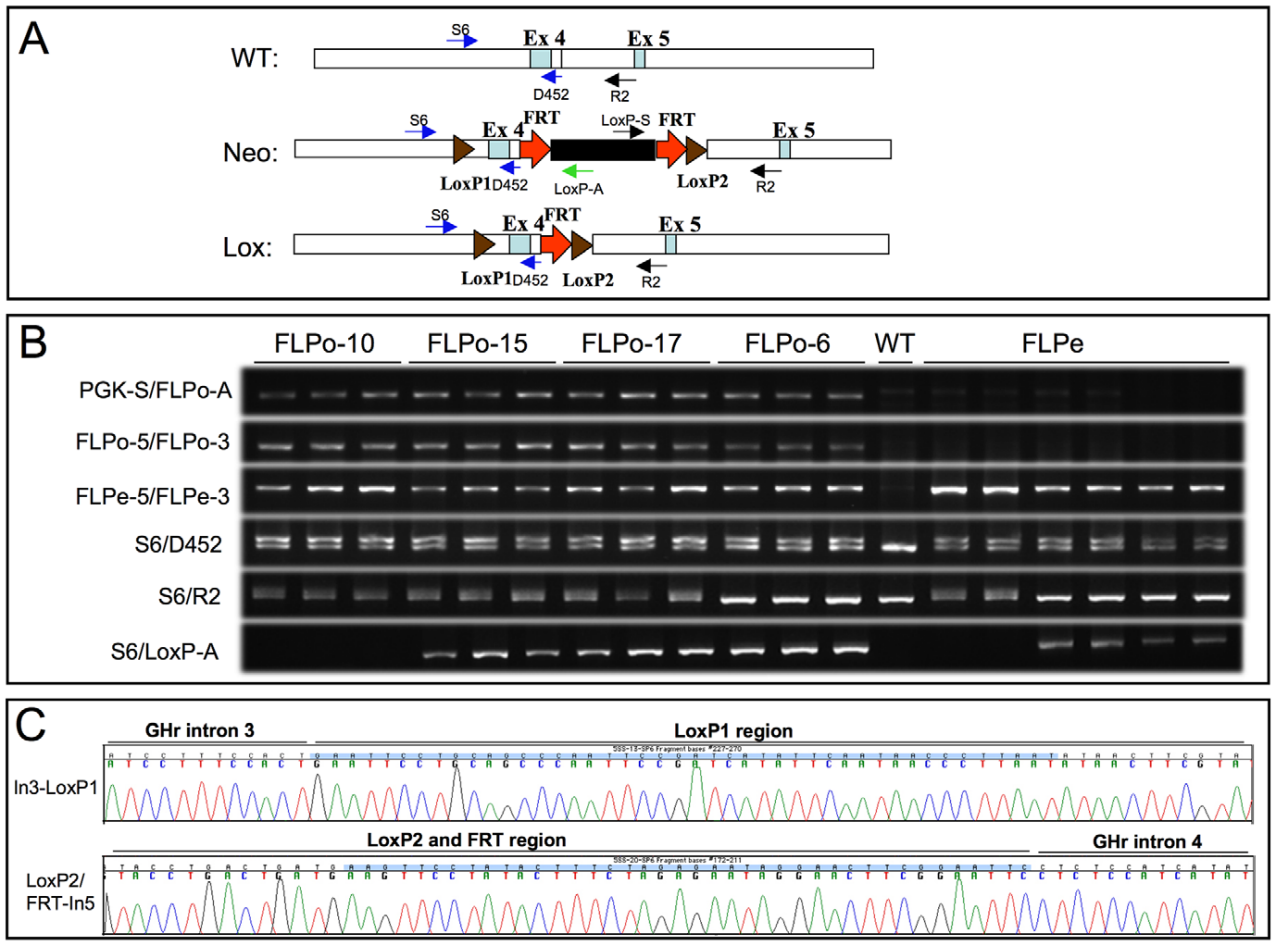
FLPo-mediated recombination in vivo. (A) Schematic diagram of gene structures of wild type growth hormone receptor GHR locus (shown are exons 4–5 flanking region), Neo allele (containing FRT/LoxP cassettes) and Lox allele (indicating the removal of Neo cassette by FLPo recombination). PCR primers used for genotyping are indicated. Red arrow: FRT sites; Brown triangle: LoxP sites. (B) PCR genotyping of F1 progeny from crosses made with 4 FLPo founder lines or FLPe transgenic mice. Wild type (WT) mice were used as control. Genomic tail DNA was used as PCR templates. PGK-S/FLPo-A and FLPo-5/FLPo-3 are specific primers for the amplification of FLPo transgene. FLPe-5/FLPe-3 primers for both FLPo and FLPe transgenes, S6/LoxP-A specific primers for the Neo cassette, S6/D452 primers distinguish between WT, Neo and the Lox alleles. S6/R2 primers distinguish between Lox and WT alleles. (C) Sequence of Lox allele in the junctions of LoxP sites.

To determine the efficiency of FLPo-mediated recombination, 4 heterozygous *PGK-FLPo* transgenic founder mice (FLPo10, FLPo17, FLPo15 and FLPo6) were crossed with mice that carry the FRT-flanked PGK-Neo cassette (GHR^Neo^/+) in intron 5 of the growth hormone receptor GHR gene (Fig. 2A). As a comparison, GHR^Neo^/+ mice were also crossed with the FLPe deleter mice. In the F1 generation, 4 genotypes of mice were expected: wild type (+/+); PGK-FLPo/+; GHR^Neo^/+ and GHR^Neo^/PGK-FLPo. FLPo-mediated recombination can only be evaluated in mice carrying both the FLPo transgene and the Neo cassette. In these mice, if FLPo exerts its enzymatic activity in all cells and results in a complete removal of the FRT-flanked Neo cassette through recombination-based FRT site excision, the genotype of these double transgenic mice should become GHR^Lox^/PGK-FLPo. However, if FLPo exhibits partial enzymatic activity, it will result in a mosaic of cells retaining the Neo cassette or lacking Neo cassette. In this case, a chimera of GHR^Neo^/GHR^Lox^/PGK-FLPo will be detected. The ratio between GHR^Lox^ and GHR^Neo^ can be used for estimation of the efficiency of FLPo-mediated recombination in vivo.

As shown in Fig. 2B, the FLPe primer set can amplify both FLPe and FLPo transgenes whereas the FLPo primer set specifically amplifies the FLPo transgene. All chosen samples carry the FLP transgene (either FLPo or FLPe) for the simplicity of analysis. S6/LoxP-A primer set specifically amplifies the intact FRT-Neo cassette (PCR positive for this primer set, hence, can be scored as “Neo” allele). S6/D452 primer set can distinguish Neo and Lox allele from wild type (990 bp and 840 bp, respectively) and S6/R2 primer set can differ between wild type allele and Lox allele (817 bp and 1019 bp, respectively; Neo allele cannot be amplified under our PCR conditions). The upper band (1019-bp) is scored as a “Lox” allele, and was confirmed by subsequent sequencing (Fig. 2C). The Lox allele can only be produced when FLP-mediated recombination occurs. In F1 pups derived from crosses with FLPo15, FLPo17 and FLPo6 transgenic mice, none had the Neo-cassette removed completely, among the mice carrying the FLPo transgene (Table 1 and Fig. 2B). Partial removal was seen with FLPo15 and FLPo17 transgenic mice as indicated by the presence of both Neo and Lox alleles, indicating mosaic recombination. In contrast, when crosses were made with FLPo-10 transgenic mice, all F1 pups carrying the FLPo transgene have Lox alleles, but not the Neo allele (Table 1 and Fig. 2B). This indicates a 100% removal of FRT-flanked Neo cassette by FLPo-mediate recombination in all cells and FLPo-10 mice can be used as a very efficient deleter mouse line. FLPe deleter mice showed less than 10% complete recombination and less than 20% partial recombination as shown by the chimera of Neo and Lox allele (Table 1 and Fig. 2B).

**Table 1:** Recombination efficiency of different FLP transgenic lines.

Line	Number of double-transgenic mice	Number of mice without the Neo-cassette	Number of mosaic mice
FLPe	24	2	4
FLPo-10	22	22	0
FLPo-15	11	0	7
FLPo-17	10	0	9
FLPo-6	9	0	0

**Figure 3:**
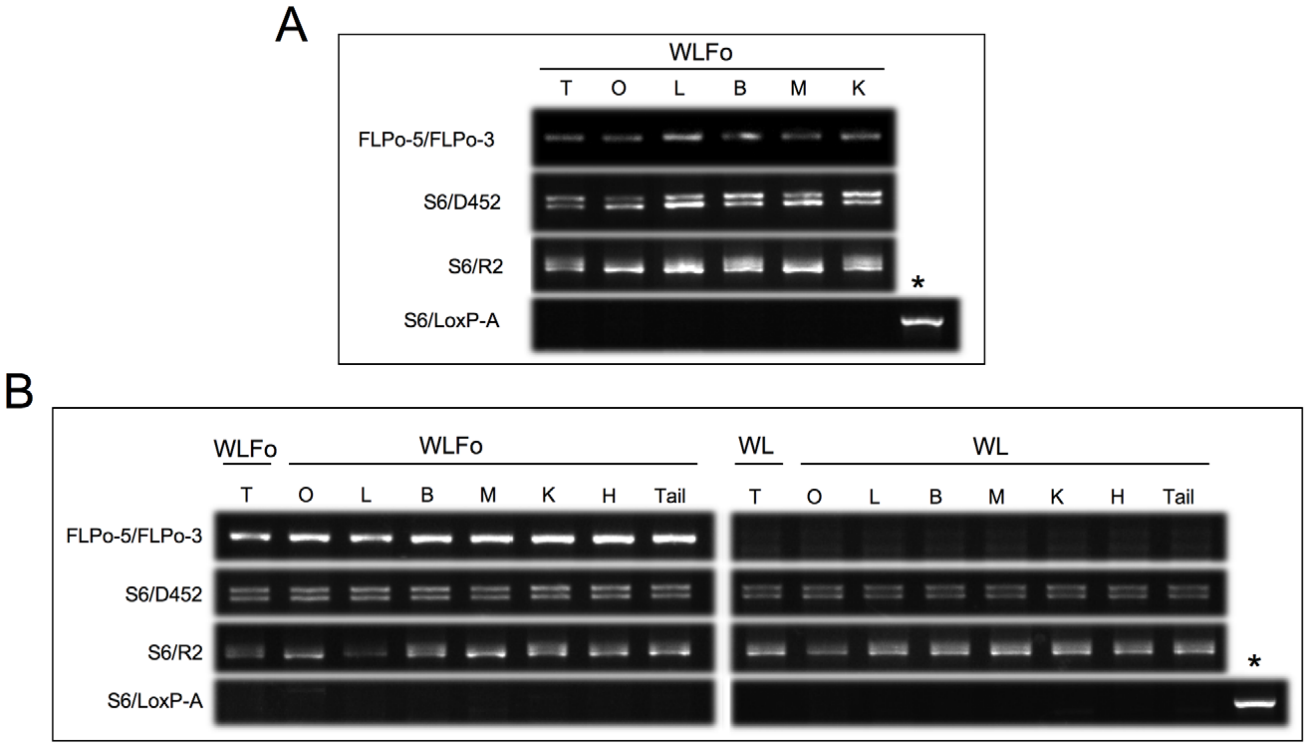
FLPo expression and activity in different tissues. (A) PCR genotyping of Neo cassette removal in different tissues of F1 offsprings. (B) PCR of different tissues [testis(T), ovary(O) liver(L) brain(B)muscle(M) kidney(K) heart(H)] of 4 WL mice with (WLFo) or without (WL) the FLPo transgene. Using primer sets as specified in Fig. 2. we found recombination in all tissues when the FLPo transgene was present. * indicates PCR positive control using S6/LoxP-A primer set for amplification of Neo cassette.

The FLP activity in the tail tips may not represent recombination in the germline. Since only removal of Neo cassette occurring in the germline can be transmitted permanently to the next generation, we first sought to examine whether removal of the Neo cassette also took place in other tissues in F1 generation. Of note, a FRT-flanked GFP reporter line for FLP mediated recombination would be optimal to address this question. However, such a reporter line is not available commercially, therefore, we performed PCR in various tissues. As shown in Fig. 3A, removal of the Neo cassette was clearly observed in testis, ovary, liver, brain, muscle and kidney. Next, we analyzed whether removal of Neo cassette is independent of co-inheritance of the FLPo transgene in the F2 generation. As shown in Table 2, removal of Neo cassette was observed in all 10 floxed mice independent of the status of FLPo activity in F2 generation (as only 3 mice segregated the FLPo transgenes in the F2). In contrast, crosses made with the FLPo-15 line, that only showed mosaic removal of Neo cassette in F1 generation, yielded 12 out of 14 flox mice still containing the Neo cassette, indicating limited removal of the Neo cassette in the F2 generation. To further confirm the complete removal of Neo cassette by FLPo-10 line, we also performed similar PCR genotyping in other tissues including gonads and obtained similar data as obtained with tail DNA (Fig. 3B).

**Table 2:** Recombination analysis of F2 generation.

Line	FLPo-10	FLPo-15
Number of mice in the F2 generation	13	17
Number of mice in the F2 generation Carrying the *Flox* allele	10	14
Number of mice in the F2 generation Carrying the *Flox* allele and the Neo cassette	0	12
Number of mice in the F2 generation Carrying the *Flox* allele without the Neo cassette	10	2
Number of mice in the F2 generation carrying the FLPo transgene.	3	4

The recombination efficiency for each line is summarized in Table 1 and 2. We thus calculated the efficiency of Flp recombination in FLPo-10 line as 100% (22 out of 22 mice). Similarly, the partial recombination was found in FLPo-15, 17 and control FLPe lines. No recombination occurred in progenies derived from FLPo-6 line. It could be due to positional effects that result in differential expression levels of the FLPo transgene. We confirmed the successful recombination by FLPo-10 transgenic line in several other target mice carrying FRT-flanked Neo in various loci. Furthermore, all FRT-cassette removed mice we tested can maintain their neo-deleted status in the next generation and 100% germline-transmitted FLPo recombination was observed in subsequent six generations of backcrosses.

## Discussion

We report here the generation of highly efficient FLPo deleter mice in C57BL/6 background. It exhibited 100% complete removal of FRT-flanked Neo cassette in the F1 progeny mice carrying both FLPo transgene and the Neo cassette in different genetic loci. Importantly, the complete removal of Neo cassette was maintained in F2 generation independently of FLPo activity. In the subsequent six generations of crosses with the wild type mice, 100% germline transmission of Lox allele (after Neo cassette removed by FLPo) was observed in each generation in the absence of FLPo transgene, indicating the superior recombination efficacy of our newly developed FLPo deleter mice.

The predominant use of conditional knockout in mouse genetic studies demands additional effective site-specific recombinase other than Cre for more delicate and efficient gene manipulation. Although FLP was the first site-specific recombinase used in genetic engineering of higher eukaryotes, successful site-specific recombination in mammalian cells and mice was first achieved with and mostly involved Cre, largely due to its highly efficient recombination activity in mammals. Despite initial reports that the efficacy of Flp recombination could be as high as 90% in mouse ES cells (AK-7) [14] and 30–78% in CCE ES cells [4], FLP exhibits low recombination activity in germ cells [15]. For example, Meyers et al (1998) reported that complete FLP-mediated recombination was not observed in all cells of the F1 pups that inherited both Neo cassette and Flp transgene although some of the mosaic animals transmitted the recombined allele (which lacks the neo cassette) to their F2 progeny [16].

The improved version of FLP, FLPe exhibits 4–10 fold enhanced thermal stability at 37°C [6]. Nonetheless, FLPe was reported to still show limited recombination rate [7] and 10% efficiency of Cre on chromatin targets [11]. As a consequence, deleter mice generated with this improved FLPe are much less efficient compare to Cre deleter mice in terms of mediating recombination in vivo. Surprisingly, no data on FLP-mediated recombination rate in F1 generation has been reported with several available FLP deleter mice. For example, Mishina et al reported 71% germline-transmitted FLP recombination in F2 generation in C57BJ/6J background. In the original paper describing the first generation of FLPe deleter mice in B6SJLF2 background, 100% germline-transmission of FLP recombination was observed in F2 generation [17]. Farley et al generated FLPe deleter mice in the ROSA26 locus in 129/SvJaadSor background, which showed complete recombination in half of all double-transgenic F1 progeny (carrying both FLPe and Neo cassette) though many of them transmitted recombined transgenes to their progenies [8]. Hiroaki Kanki et al reported high recombination rate in two CAG-NLS-FLPe transgenic mice [10]. However, the number of mice examined was very limited (at most 6 mice).

In contrast, the mouse codon-optimized FLPo gene has been shown to be highly active in mouse ES cells, resulting in comparable recombination efficiency as Cre [12]. Although it was not shown how codon-optimized FLPo improved the recombination efficiency in ES cells and the comparison of the recombination efficiency between FLPo and FLPe might be flawed (since gene copy number could be different in the FLPo and FLPe stable clones used in the comparison study), FLPo exhibited higher recombination efficiency than FLPe in human cells when both transiently expressed. During the process of making conditional knockout in human colon cancer cell line HCT116, while no removal of Neo cassette was observed in 24 clones carrying the FLPe transgene (either under CAG or CMV promoter), 100% clones carrying the FLPo transgene (under PGK promoter) become G418-sensitive (unpublished data). Such superior recombination efficacy of FLPo remains in the FLPo deleter mice. In a comparision with FLPe deleter mice, FLPo mice exhibited 100% complete removal of Neo cassette in all F1 progeny whereas FLPe mice showed excision in only 10% of the F1 offspring. Due to the low frequency, progeny with complete removal of Neo cassette were often not detected until second or third round of mating, which will take additional 3–6 weeks. However, due to different promoters used in FLPo and FLPe deleter mice and the complication of positional effect, it is hard to conclude whether improved recombination efficacy of FLPo over FLPe is due to codon-optimization or due to gene copy number. Additionally, there is a possibility that chromosomal rearrangement could have occurred during passage in the FLPe mice we obtained, resulting in loss of gene copy number of the functional FLPe gene. In addition, FLPe and FLPo might exhibit differential expression levels in different tissues. Surprisingly, a recent report by Kondo et al showed that FLPe exhibits lower recombination efficiency than the original wild type FLP on a molar basis even at 37°C [18]. However, the steady-state of FLPe protein level is 3 times of wild type FLP in mammalian cells, indicating that the higher recombination efficiency observed with FLPe is largely due to its enhanced thermostability in the expense of lower enzymatic activity. They also showed that a human codon-optimized FLPe (hFLPe) is more efficiently translated in mammalian cells than FLPe and hence, resulted in 10-fold higher protein yield when transiently expressed under the same ectopic promoter [18]. Since FLPo and hFLPe are identical at amino acid level and both are codon-optimized, it is likely that FLPo is also more efficiently translated than FLPe.

In summary, our FLPo deleter mouse line is valuable for gene manipulation and will facilitate the general process of generation of conditional knockout mice. This line will be donated to The Jackson Laboratory. For information on obtaining the animals, please refer to the URL: hrttp://www.jaxsonlab.

## Materials and Methods

### Animals

All mouse models used in the study were bred on a C57B6/J background. Animal care and maintenance were provided through the Mount Sinai School of Medicine AAALAC Accredited Animal Facility. All procedures were approved by the Animal Care and Use Committee of the Mount Sinai School of Medicine.

### Materials

pPGKFLPobpA plasmid was constructed by Raymond et al (2008) and purchased from Addgene (Addgene plasmid 13793) [12]. The FLPe transgenic mice B6.Cg Tg(ACTFLPe)9205Dym/J (JAX Mice and Services Stock number 005703) were purchased from the Jackson Laboratory [12].

### Generation and Characterization of Transgenic Mice

The 2.2-kb Sal I/Not I fragment from the pPGKFLPobpA plasmid was purified by QIAquick Gel Extraction Kit (QIAGEN Inc. CA, USA). The linearized DNA solution (2 ug/ml) was microinjected into pronuclei of fertilized one-cell embryos of C57BL/6N mice. Injected embryos were transplanted into the pseudopregnant mice. Breeding and maintenance of mice were performed under institutional guidelines. Mice were fed ad libitum with standard laboratory chow and water under a 12-h light/dark cycle. FLPo transgenic mice were genotyped by PCR with a primer set: PGK-P and Flpo-A or Flpo 5 and Flpo3. (Information of primer sequences can be obtained upon request).

### Analysis of Flp Recombination In Vivo

The heterozygous *PGK-FLPo* transgenic mice FLPo10, FLPo17, FLPo15 and FLPo6 were crossed with mice that carry the FRT-flanked PGK-Neo cassette. The F1 pups from this mating were genotyped for the PGK-FLPo transgene and the FRT-Neo cassette. F1 progeny mice carrying both the FLPo transgene and the Lox allele were backcrossed to wild-type mice to remove the FLPo transgene. Genomic DNA of F1 and F2 progeny was subjected to PCR analysis. S6/D452 primer set was used for distinguish wild type from Neo and Lox alleles (840 bp and 990 bp, respectively). Primer set S6/R2 was used for distinguish wild type and Lox allele (1019 bp and817 bp, respectively). Genomic DNA isolation from tissues and tail is following the standard protocol.

### FLPo Expression Analysis by RT-PCR

Total RNA from tissues was extracted using TRIzol reagent according to the manufacturer's instructions (Invitrogen Corp., Carlsbad, CA, USA). RNA integrity was verified using Bioanalyzer (Agilent Technologies 2100 Bioanalyzer-Bio Sizing, Version A.02.12 SI292). One ug of RNA was reverse-transcribed to cDNA using oligo(dT) primers with RT-PCR kit according to the manufacturer's instructions (Invitrogen Corp., Carlsbad, CA, USA). RT-PCR was performed and b-actin was used as control.
